# Towards Replacing Titanium with Copper in the Bipolar Plates for Proton Exchange Membrane Water Electrolysis

**DOI:** 10.3390/ma15051628

**Published:** 2022-02-22

**Authors:** Andrea Kellenberger, Nicolae Vaszilcsin, Delia Duca, Mircea Laurentiu Dan, Narcis Duteanu, Svenja Stiber, Tobias Morawietz, Indro Biswas, Syed Asif Ansar, Pawel Gazdzicki, Florian J. Wirkert, Jeffrey Roth, Ulrich Rost, Michael Brodmann, Aldo Saul Gago, K. Andreas Friedrich

**Affiliations:** 1 Faculty of Industrial Chemistry and Environmental Engineering, Politehnica University Timisoara, Piata Victoriei No. 2, 300006 Timisoara, Romania; nicolae.vaszilcsin@upt.ro (N.V.); duca.delia@gmail.com (D.D.); mircea.dan@upt.ro (M.L.D.); narcis.duteanu@upt.ro (N.D.); 2 Institute of Engineering Thermodynamics, German Aerospace Center, Pfaffenwaldring 30-40, 70569 Stuttgart, Germany; svenja.stiber@dlr.de (S.S.); tobias.morawietz@dlr.de (T.M.); indro.biswas@dlr.de (I.B.); syed-asif.ansar@dlr.de (S.A.A.); pawel.gazdzicki@dlr.de (P.G.); andreas.friedrich@dlr.de (K.A.F.); 3 Faculty of Science, Energy and Building Services, Esslingen University of Applied Sciences, Kanalstraße 33, 73728 Esslingen am Neckar, Germany; 4 Westfälisches Energieinstitut, Westfälische Hochschule University of Applied Sciences, Neidenburger Str. 43, 45897 Gelsenkirchen, Germany; florian.wirkert@w-hs.de (F.J.W.); jeffrey.roth@w-hs.de (J.R.); ulrich.rost@w-hs.de (U.R.); michael.brodmann@w-hs.de (M.B.)

**Keywords:** PEMWE, water electrolysis, cost reduction, bipolar plate, corrosion resistance, coatings

## Abstract

For proton exchange membrane water electrolysis (PEMWE) to become competitive, the cost of stack components, such as bipolar plates (BPP), needs to be reduced. This can be achieved by using coated low-cost materials, such as copper as alternative to titanium. Herein we report on highly corrosion-resistant copper BPP coated with niobium. All investigated samples showed excellent corrosion resistance properties, with corrosion currents lower than 0.1 µA cm^−2^ in a simulated PEM electrolyzer environment at two different pH values. The physico-chemical properties of the Nb coatings are thoroughly characterized by scanning electron microscopy (SEM), electrochemical impedance spectroscopy (EIS), X-ray photoelectron spectroscopy (XPS), and atomic force microscopy (AFM). A 30 µm thick Nb coating fully protects the Cu against corrosion due to the formation of a passive oxide layer on its surface, predominantly composed of Nb_2_O_5_. The thickness of the passive oxide layer determined by both EIS and XPS is in the range of 10 nm. The results reported here demonstrate the effectiveness of Nb for protecting Cu against corrosion, opening the possibility to use it for the manufacturing of BPP for PEMWE. The latter was confirmed by its successful implementation in a single cell PEMWE based on hydraulic compression technology.

## 1. Introduction

Hydrogen produced by water electrolysis represents a green alternative to hydrogen obtained from fossil fuels. However, only about 4% of the world’s hydrogen production is obtained by electrolysis because its cost is still non-competitive with classical methods [1,2]. Currently, there are two types of industrial electrolysis technologies for hydrogen production: alkaline water electrolysis (AWE) and proton exchange membrane water electrolysis (PEMWE). For decades, the former has been used at a large scale for hydrogen production, but the efficiency is low, and its range of operation is limited. Alternatively, PEMWE has the advantage of having higher efficiency than AWE and the ability to conduct electrolysis at high current densities and pressures, reducing the capital costs. Moreover, PEMWE can operate at variable current densities, making them suitable for connection with renewable energy sources [2,3,4]. The main disadvantage of the PEMWE consists in the harsh environment inside an operating electrolyzer, in particular the high temperature and electrochemical potential, as well as strong acidic conditions at the anode side, which implies significant corrosion problems of the stack components [5,6] and also degradation and durability issues of the membrane-electrode assembly [7,8,9] due to membrane thinning and both anode and cathode catalyst dissolution [10,11]. Titanium is currently used to manufacture bipolar plates (BPP) and porous transport layers (PTL) at the anode side, involving high costs for the PEM electrolyzer manufacturing process [12]. Highly corrosion-resistant materials are also essential for the anode electrocatalysts, which are usually based on precious metal oxides [13], such as unsupported Ir-oxide [14,15], supported Ir-oxide [16,17,18,19], supported Ir and Ru nanoparticles [20], or IrSn-oxide [21].

A technical and economic analysis by Buttler and Spliethoff [22] on water electrolyzers used nowadays reveals that the costs of PEMWE manufacturing, installation, operation, and maintenance are almost double compared to AWE. Researchers focus on reducing the capital cost of the PEM electrolyzers by reducing the amount of expensive materials, aiming to bring the costs closer to the alkaline technology [23]. One of the key components in PEMWE are the BPPs, since they must fulfill several roles: assuring electrical contact between cells in the stack and water distribution to the anode. They are also one of the most expensive components of a PEM electrolyzer stack [24,25] since they need to be manufactured from Ti to withstand the high potentials and the highly corrosive environment at the anode side. The approach for reducing costs of BPPs is the use of less expensive base metals that are easier to manufacture than Ti, protected with a highly conductive and corrosion-resistant coating. We have previously demonstrated, for the first time, that stainless steel can be used as base material for BPPs by applying an electrically conductive and corrosion-resistant bi-layer coating, which consists of a 50–60 µm Ti layer deposited by plasma spraying and a 1.5 µm Pt layer deposited by magnetron sputtering physical vapor deposition (PVD) [26,27]. The Pt layer is necessary to maintain a low contact resistance because the Ti passivation leads to a semi-conducting layer of TiO_2_ on its surface. Moreover, we have also demonstrated that Pt can be successfully replaced with corrosion resistant and highly conductive coatings of Nb [28]. Similarly, Nb and Nb/Ti coatings have also been used for corrosion protection of stainless-steel bipolar plates in PEM fuel cells [29,30,31].

The excellent corrosion resistance properties of Nb are due to its ability to passivate spontaneously in contact with oxygen from air or aqueous environments, by forming a thin, highly adherent and stable passive oxide layer on its surface. The thickness of this oxide layer is 2–4 nm and it is extremely difficult to remove from the metal surface [32,33]. The corrosion resistance of Nb can be further increased by electrochemical formation of passive oxide films on its surface. The chemical composition of the anodic oxide film has been found to depend on the presence of the natural air-formed oxide film and consists of more or less stable oxides, such as NbO, NbO_2_, and Nb_2_O_5_ [32,33]. During anodic polarization, NbO_2_ is irreversibly oxidized to Nb_2_O_5_ and Nb is reversibly oxidized to NbO. Raman spectra revealed that the passive film formed at higher voltages consists primarily of Nb_2_O_5_ [33]. It has also been found that Nb shows significantly greater corrosion resistance than Ti in the presence of increased concentrations of fluoride ions, which are released from the membrane, due to its stable passive film formed mainly by Nb_2_O_5_ with a thickness of 4–8 nm determined by X-ray photoelectron spectroscopy (XPS) [34].

Previously, we developed an Nb/Ti coating for stainless steel BPP solving the challenges of removing precious metal coatings and replacing Ti as base material [27]. However, the Nb/Ti coating had to be produced in two steps since the thin coating of Nb was not sufficient to protect stainless steel against corrosion, thus the plasma sprayed coating of Ti was necessary. However, ideally one plasma-sprayed coating of Nb would be preferable. Moreover, stainless steel does not possess excellent thermal and electrical properties similar to copper. Because of its properties and lower cost compared to titanium, Cu would be an ideal material to manufacture BPPs. However, its utilization in an electrochemical device that has such an aggressive environment, full of water, with high oxygen content and traces of fluoride ions, low pH, and temperatures of 80 °C or above, is unthinkable. It has been shown that 5 ppm Cu^2+^ causes significant performance decay without a subsequent recovery, as Cu^2+^ tends to adsorb and remain in the membrane [35]. 

In this work we have managed to implement a material with zero tolerance, such as Cu for the manufacturing of BPPs for PEMWE by applying a complete dense single-step coating Nb by plasma spraying on its surface. The influence of coating thickness, given by the number of deposited layers, on the corrosion resistance properties is investigated by electrochemical methods. The Nb coating fully protects the Cu substrate against corrosion in a simulated environment of PEMWE and it was successfully implemented in a complete cell based on hydraulic compression technology. 

## 2. Materials and Methods

### 2.1. Deposition of Coatings by VPS

Nb-coatings were deposited via vacuum plasma spraying (VPS) on 0.5 mm thin copper plates. To prevent contamination of the coating, the copper substrate was cut by water cutting to 15 mm discs, which were still held by a frame for the coating process. The substrate was preheated to 250 °C before powder deposition. The feedstock powder of Nb (H.C. Starck nowadays FST Flame Spray Technologies, Duiven, The Netherlands) had a particle size of 45 µm. To achieve the needed plasma enthalpy of 21.3 MJ kg^−1^, the gas flow rates of Ar, N_2_, and H_2_ were carefully chosen. Three different coatings were produced by varying the iterative number of 8, 16, and 32 coating runs, by means of the number of times that the spraying nozzle moves over the copper substrate. The particular coatings result in different thicknesses of 30 µm, 70 µm, and, respectively, 130 µm (as determined from cross-section scanning electron microscopy images). For the coating, the torch sweep rate was 350 mm s^−1^. Samples were denoted as NbCu8L, NbCu16L, and NbCu32L, referring to the number of deposited layers.

After coating, the remaining pores were sealed using an epoxy-resin and a hardener, with the chemical composition given in Table 1. The resin was deposited on top of the VPS-coating and a vacuum of 150 mm Hg was applied for less than 5 min. Finally, the samples were dried in an oven over night at 65 °C. To achieve a shiny metallic surface, the NbCu samples were grinded with sand paper of 240P grain size and, in a final step, polished with sand paper of 2400P grain size. Physico-chemical characterization and corrosion testing was performed with the produced samples. 

### 2.2. Physico-Chemical Characterization of Coatings

The structure and morphology of the niobium coatings has been characterized by field emission scanning electron microscopy (FE-SEM) using a QUANTA FEG 250 microscope (FEI, Hillsboro, OR, USA) and the elemental composition was determined by energy dispersive X-Ray analysis (EDX, EDAX Inc., Mahwah, NJ, USA) before and after corrosion testing. FE-SEM images were taken using the backscattered electrons detector (BSED), at an accelerating voltage of 15 or 20 kV and with a working distance of 10 mm. The surface images were further analyzed using ImageJ, a public domain software [36], to approximate the area fraction corresponding to niobium and resin.

Interfacial contact resistance (ICR) versus compaction force measurements were performed before the corrosion test to evaluate the conductivity of the coatings. This technique was firstly developed to determine ICR of BPP in PEM fuel cells, but it has also been used for BPP in PEM water electrolyzers [6,26,27,28]. The uncoated BPP side was first polished with SiC (grain size 2400) and afterwards cleaned with 0.5 M H_2_SO_4_ to remove the oxide formed in air. The Nb coated side was polished in two steps with SiC, first with a grain size of 2400 and second with a grain size of 4000. For the measurement, the Nb- coated side was put in contact with a gas diffusion layer (GDL) made of untreated carbon paper (Spectracarb^TM^ 2050A-6060, 1524 µm thick) and placed between two gold-coated copper plates. This sandwich-like assembly was placed between two insulating poly(methyl methacrylate) (PMMA) plates and introduced into a hydraulic press. The ICR measurements were performed by applying a direct current of 1A between the two gold-plated copper plates using a power source followed by measuring the voltage with a precision multimeter Picotest M3510A (Phoenix, AZ, USA). The compaction force varied in steps from 50 to 1500 N, which corresponds to a compaction pressure of approximately 30 to 800 N cm^−2^. The ICR of the Nb coating was calculated from an electrical equivalent circuit of resistors connected in series, each resistor corresponding to a contact interface. 

### 2.3. Half-Cell Corrosion Testing

All electrochemical measurements were performed using an Autolab PGSTAT 302N potentiostat/galvanostat (Metrohm Autolab, Utrecht, The Netherlands) and a water jacket three-electrode configuration corrosion cell. The working electrode consisted of 15 mm disc-shaped copper plates coated with a protective layer of niobium. The reference electrode was Ag/AgCl (3M KCl) placed in close vicinity of the working electrode via a Luggin capillary, and the counter electrode was a Pt gauze. The configuration of the electrochemical cell prevents the copper substrate from getting in contact with the test solution, and only an area of 1 cm^2^ of the coated surface is exposed to the test solution. Before conducting corrosion tests, the sample’s surface was mechanically polished with SiC paper grit 4000. Corrosion tests were performed at two different pH values, 2 and 1.4, corresponding to 0.005 M and 0.05 M of H_2_SO_4_ solution, respectively, at 90 °C in O_2_-saturated solutions to simulate the anode side of the PEM electrolyzer environment under operating conditions and in the presence of 0.1 ppm fluoride ions to simulate conditions due to the proton exchange membrane degradation via fluoride release. Corrosion parameters, such as corrosion potential *E*_corr_, corrosion current *j*_corr_, and corrosion rate *v*_corr_ have been determined for each sample before and after an accelerated stress test (AST), achieved by polarizing the samples at a constant potential of 2 V for 6 h. Chronoamperometric curves have been recorded during polarization at a constant potential of 2 V for 6 h. Electrochemical impedance spectroscopy (EIS) measurements were performed at open circuit potential, before and after the accelerated stress test, in the frequency range from 10^−3^ to 10^5^ Hz and AC voltage amplitude of 10 mV rms. For each spectrum, 60 points were collected with a logarithmic distribution of 10 points per decade. The experimental EIS data were fitted to the equivalent electrical circuit (EEC) by a complex non-linear least squares Levenberg–Marquardt procedure using the ZView 3.0 software (Scribner Associates, Inc., Southern Pines, NC, USA). 

### 2.4. Post-Test Analytic

The elemental and chemical compositions of Nb coatings after the corrosion tests were investigated with X-ray photoemission spectroscopy. In combination, the bombardment with accelerated Argon ions was used to, stepwise, etch the surface to lay open the layer composition. The analysis was performed with a hemispherical electron analyzer (Thermo Scientific ESCALAB 250, Waltham, MA, USA) in a vacuum chamber of a base pressure of 2 × 10^−10^ mbar. Samples were excited with a conventional Al Kα source, and a monochromated Al Kα source for specific high-resolution data (photon energy of 1486.6 eV). Spectra were energy calibrated with reference to the Ag3d3/2 signal of a clean, etched, silver surface. Numerical peak fitting of the recorded spectra was performed using convoluted Gaussian/Lorentzian peak shapes (Unifit 2016) [37]. Depth profiling by Ar^+^ ion bombardment was performed with a scanning off-axis ion source (Thermo Scientific EX05) at an argon pressure of ~2.5 × 10^−8^ mbar and with an average sample current density of 0.2 µA/mm².

Atomic Force Microscopy (AFM, Bruker Nano Surfaces Inc., Karlsruhe, Baden-Württemberg, Germany) measurements were also performed after the corrosion measurements. The oxide layer was removed with sandpaper (4000) on part of the sample and then cleaned with ethanol before measurements. The sample was attached to a 12 mm steel disc with conductive double-sided tape. AFM measurements were performed with a Bruker Multimode 8 AFM in PF TUNA conductive tapping mode. A conductive diamond tip was used, and a bias voltage of 3 V was applied between the AFM tip (Conductive diamond coated, Nanosensors) and the sample. 

### 2.5. PEMWE Test

Nb-coated Cu pole plates were implemented in a single-cell PEMWE using the hydraulic compression of the cell components. The concept of hydraulic cell compression has already been realized using a pressure housing with flexible pockets [38,39]. PEM electrolyzer cells are placed into these pockets and the pressure inside this stack is directly applied to the bipolar plates of every single cell. This is a promising approach for PEM electrolyzer operation, as a homogeneous pressure distribution is guaranteed independent of the cell size. Hence, a homogeneous current distribution can be achieved and both thermal and mechanical stresses can be minimized [40].

The PEM electrolyzer cell for evaluating the use of Nb coating on copper pole plates had an active area of 25 cm^2^. The interconnecting components used on the anode side of the cell were the Nb-coated Cu pole plate as well as a Ti compound PTL (GKN Sinter Metals), while for the cathode side a commercial, carbon-based PTL (Spectracarb™ 2050A-6060) and a coated copper pole plate was used. Membrane electrode assemblies (MEAs) with an Ir-based anode, Pt-based cathode, and chemically stabilized Nafion 115 membrane were used for all electrochemical tests. Polarization curves up to 2 A cm^−2^ following the JRC protocol [41] were performed. All measurements were performed at 80 °C and 8 bar hydraulic pressure. 

## 3. Results and Discussion

### 3.1. Physico-Chemical Characterization of Coatings

**Figure 1:**
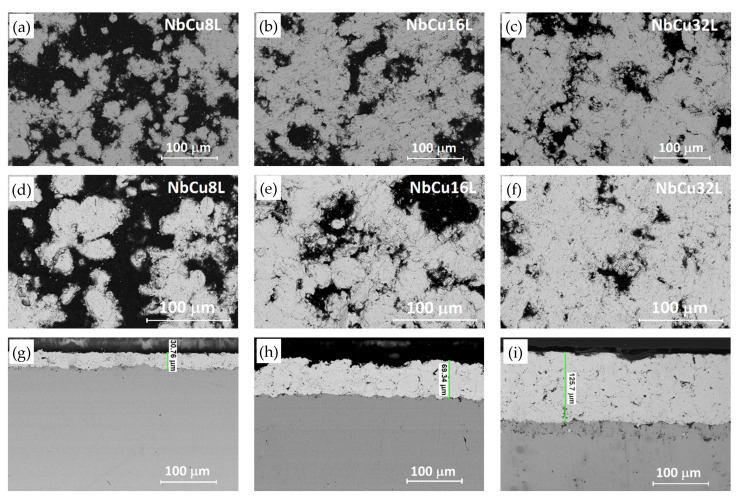
FE-SEM images of Nb coatings on Cu: (**a**–**c**) surface before corrosion, (**d**–**f**) surface and (**g**–**i**) cross-section after corrosion test in 0.05 M H_2_SO_4_ + 0.1 ppm F^−^ (pH = 1.4).

Nb coatings on Cu were investigated by FE-SEM and EDX before and after corrosion tests. Figure 1 shows FE-SEM images of the coatings as deposited, as well as surface and cross-sectional images after performing the corrosion test at pH = 1.4. Since FE-SEM images were taken with a back-scattered electron detector, Figure 1 shows a contrast between areas with different chemical compositions. Bright areas correspond to heavy elements, i.e., niobium, while dark areas correspond to lighter elements, i.e., C, O, and N, originating from the epoxy-resin that fills the pores. FE-SEM images indicate a surface roughness of the coatings, which reduces with the increase in the number of deposited layers. FE-SEM images were processed using an image analysis software and the surface fraction of Nb was approximated to be 59.2 ± 4.6% for NbCu8L, 65.7 ± 5.5% for NbCu16L, and, respectively, 71.6 ± 5.5% for NbCu32L.

According to the cross-sectional images in Figure 1g–i, the coating thickness increases with the number of deposited layers from about 30 µm for NbCu8L to 70 µm for NbCu16L and, respectively, 130 µm for NbCu32L. All coatings reveal a lamellar structure, as resulted from VPS deposition, with some micro-pores and interlamellar boundaries especially visible for the thicker coatings, but most importantly, without vertical micro-cracks or discontinuities at the interface between the coating and the substrate. A strong adhesion between the coating and the metal base is achieved by the substrate roughening and preheating prior to deposition. The mechanical properties of VPS-deposited coatings are superior to those obtained by atmospheric plasma spray, since the coatings are denser, without oxide content, and have a much greater bond strength [42]. In the case of niobium, the deposition at low pressure prevents the formation of stable or metastable niobium-oxide phases that cause brittleness of the coating [43]. Indeed, a single-phase microstructure is observed in Figure 1g–i, without inclusions of niobium-oxide phases that can cause poor interlamellar adhesion. The presence of partially melted spherical particles with diameters of 15–20 µm, non-uniformly distributed, can be recognized, but no signs of corrosion were detected underneath the coating after performing the corrosion tests. Both surface and cross-sectional images of the samples show neither apparent damage of the coatings, nor the formation of pinholes beneath the Nb coating. Results of EDX analysis of the coatings surface show an inhomogeneous composition, with about 70–80 wt% Nb and the rest corresponding to elements C, N, and O, originating from the resin and hardener used to seal the pores as well as trace amount of Si from mechanical preparation. The presence of copper was not detected by EDX analysis on the sample’s surface after corrosion tests, in agreement with cross-sectional FE-SEM images, which showed an undamaged coating.

**Figure 2:**
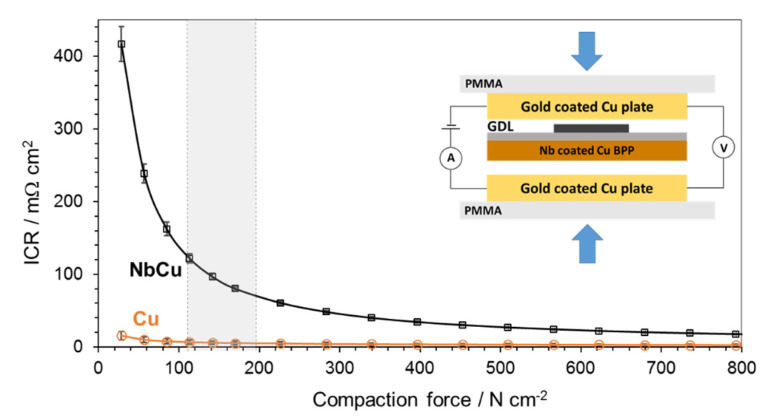
Interfacial contact resistance of Cu substrate and Nb-coated Cu pole plate at different compaction forces. Shaded area on the X-axis corresponds to the pressure range used for assembling commercial PEM electrolyzer stacks. Inset shows the experimental setup for ICR measurements.

The electrical resistance of the Nb coatings on Cu was investigated and Figure 2 shows results of ICR measurements with respect to the applied compaction pressure.

The ICR of an Nb-coated Cu plate shows an important decrease as the compaction pressure increases to a value of about 400 N cm^−2^, then it slowly decays to 25 mΩ cm^2^ at higher compaction forces. In the range of 120 to 200 N cm^−2^, which is the common pressure applied for assembling commercial PEM electrolyzer stacks [28], the ICR decreases from 115 to 70 mΩ cm^2^. Nb coatings on Cu show comparable ICR values to those obtained on metallic niobium [6,44] and Nb coatings on different substrates such as stainless steel and titanium [28]. It is noteworthy that the Nb coated Cu plate shows lower ICR values than those reported on Ti coatings [26,27,28], in fact thin Nb coatings have been used to decrease the ICR of Ti [28]. ICR values of Ti and Nb are strongly influenced by the presence of semiconducting surface oxides, which spontaneously form in air and are usually difficult to remove completely by simple mechanical polishing. Typical ICR values of Ti are around 200–300 mΩ cm^2^ at compaction forces of 120–200 N cm^−1^ [26,45], unless the surface oxides are removed by chemical etching, when the ICR values drastically decrease [6].

### 3.2. Half-Cell Corrosion Testing

All coatings were evaluated according to the same corrosion protocol, which includes the determination of open circuit potential (OCP) for 1 h, followed by electrochemical impedance measurements and potentiodynamic polarization curves. Then the samples are submitted to an accelerated stress test by polarization at 2 V for 6 h, followed again by open circuit potential, electrochemical impedance, and polarization curve measurements. Corrosion currents (*j_corr_*) and corrosion rates (*v_corr_*) were determined from the Tafel plots and polarization resistances (*R_p_*) were calculated using the Stern–Geary equation, according to Equation (1). All corrosion parameters were determined both before and after polarization at 2 V.

(1) $$R_{p}=\frac{b_{a}\cdot b_{c}}{j_{corr}\cdot 2.303\left(b_{a}+b_{c}\right)}$$

where: *R_p_* is the polarization resistance, Ω cm^2^; *b_a_* and *b_c_* represent the anodic and cathodic Tafel slopes, V and *j_corr_* is the corrosion current, A cm^−2^.

First information on the processes taking place at the electrode/electrolyte interface were obtained by recording the evolution of open circuit potential in time, presented in Figure S1 of the supporting information. The potential established in the absence of a current is determined by a combination of electrode kinetics and thermodynamics and, in time, it trends towards a steady-state value, which is equal to the corrosion potential. For metallic Nb and for Nb coatings on Cu, the OCP shifts to more positive values in time at both pH values, and the final value after reaching a near-steady-state is equal to corrosion potential values determined from the Tafel plots. The positive shift of OCP, in time, is typically attributed to the ongoing growth and stabilization of a passive film on the sample surface. It has been observed that, in less acidic solutions (pH = 2), the OCP values are less positive, while in more acidic solutions (pH = 1.4), OCP values are more positive. Also the overall shift of OCP in time from the initial to the final value is more pronounced at a lower pH. This indicates a better passivation possibility in a more acidic solution in the presence of oxygen.

**Figure 3:**
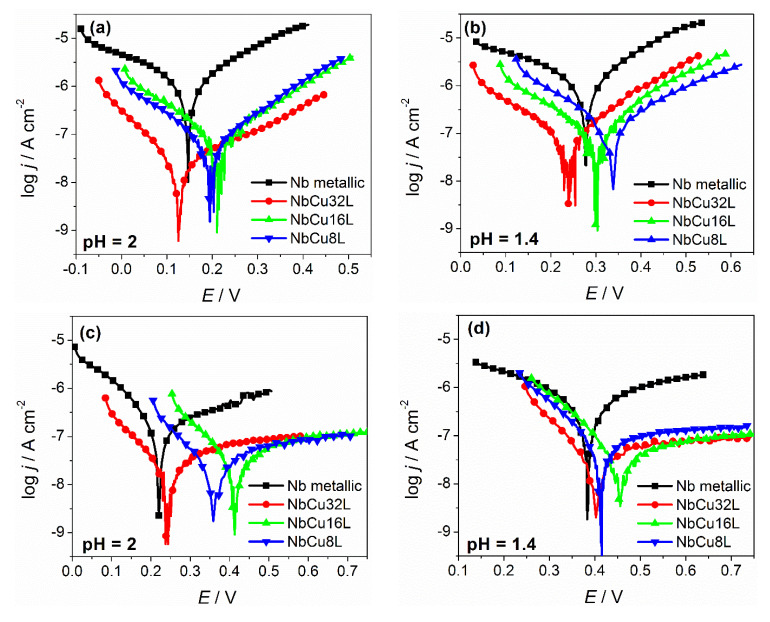

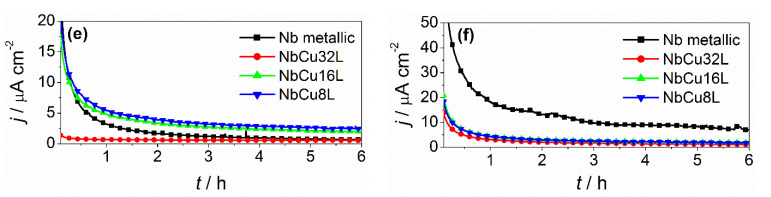
Potentiodynamic polarization curves (v = 1 mV s^−1^) measured in O_2_-saturated solutions at 90 °C for metallic Nb and Nb coatings on Cu: (**a**) before and (**c**) after AST in 0.005 M H_2_SO_4_ + 0.1 ppm F^−^ (pH = 2); (**b**) before and (**d**) after AST in 0.05 M H_2_SO_4_ + 0.1 ppm F^−^ (pH = 1.4); current transients during AST in the test solutions at (**e**) pH = 2 and (**f**) pH = 1.4.

Polarization curves of metallic Nb and of different thickness Nb coatings on Cu obtained before and after the accelerated stress test are given in Figure 3, together with current transients measured at a constant potential of 2 V, in both test solutions with pH = 2 and 1.4.

It can be observed that before polarization at 2 V, the corrosion potentials of metallic Nb and Nb coatings are in the same range, with a maximum shift of 60 mV. After polarization at 2 V, the corrosion potentials shift to more positive values for both metallic Nb and Nb coatings. Before polarization at 2 V, the cathodic and anodic branches of the Tafel plots show an almost linear behavior corresponding to the cathodic process and the anodic dissolution of niobium from a metallic state to niobium (V) according to the oxidation reaction described by Equation (2).

*2Nb + 5H_2_O→ Nb_2_O_5_ + 10H^+^ + 10e^−^* (2)

After polarization at 2 V, the slope of the anodic branch shows a pronounced increase, which is a clear indication about the formation of the protective, passive niobium-oxide layer. The anodic current density values are low, remaining almost constant over a large potential window, and correspond to the passivation current.

Table 2 and Table 3 summarize values of corrosion parameters determined from the potentiodynamic polarization curves, obtained in an acid solution at pH = 2 and 1.4, both before and after polarization at 2 V.

**Figure 4:**
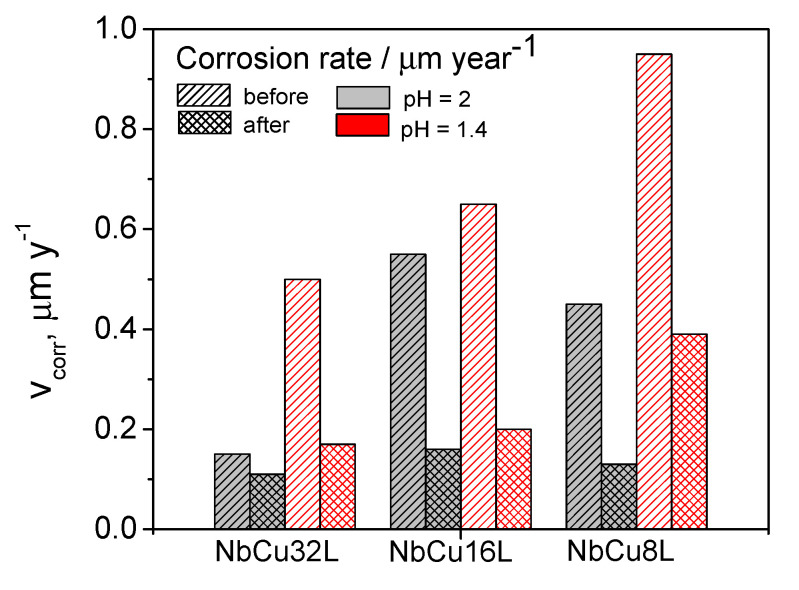
Comparison of corrosion rates of Nb coatings on Cu at pH = 2 and pH = 1.4, before and after polarization at 2 V for 6 h.

Comparing the corrosion parameters at different pH values, it can be observed that both before and after polarizations, all samples showed more positive corrosion potentials and higher corrosion currents at a lower pH. Since the cathodic reaction coupled with the anodic dissolution of niobium is the reduction reaction of dissolved oxygen, it is expected that in more acidic solutions, its equilibrium potential will shift to more positive values, which determines the corrosion potential to shift to more positive values and the corrosion rate to increase. The anodic Tafel slopes have higher values after polarization, confirming the formation of the passive oxide layer. All Nb coatings on Cu showed low corrosion current densities and high polarization resistance values. Before polarization, a lower thickness of the coating correlates with slightly higher corrosion current densities, but after the formation of the passive oxide layer, the corrosion current density becomes almost independent on the number of layers. Corrosion current density values are below 0.1 µA cm^−2^, a much lower value than the target set by the U.S. Department of Energy for the maximum corrosion current density of BPP in PEM fuel cells, of 1 µA cm^−2^ by 2020 [46]. Although there are currently no recommendations available for the corrosion resistance of BPP in PEM water electrolyzers, it is meaningful to compare this parameter to that imposed for BPP in PEM fuel cells. The corrosion currents obtained from the Tafel plots were converted to corrosion rates, expressed in µm year^−1^, assuming that uniform corrosion of Nb coating takes place. This assumption is also supported by FE-SEM images, which do not show the occurrence of localized or pitting corrosion. The corresponding values of corrosion rates are presented in Figure 4, dependent on coating thickness and solution pH.

The corrosion rates show a similar trend to that observed for corrosion currents. Before polarization, corrosion rates are higher for lower thickness coatings, i.e., a lower number of deposited Nb layers. After polarization at 2 V for 6 h and the formation of a passive layer on the sample’s surface, the corrosion rates tend to have the same value at a lower pH and are similar for samples with 32 and 16 layers at a higher pH. Nevertheless, all determined corrosion rates are below 1 µm year^−1^ for all samples, in both test solutions, and before and after polarization, indicating excellent corrosion resistance properties of the Nb coatings on Cu.

Further information about the corrosion resistance of Nb coatings is obtained during the accelerated stress test at a constant potential of 2 V for 6 h. The chronoamperometric curves given in Figure 3e,f, show an exponential decay in time, corresponding to the formation of the protective oxide layer. The current density values stabilize around 2 µA cm^−2^ for NbCu8L and NbCu16 L samples, and are even lower for NbCu32L, indicating the formation of a stable anodic oxide film. There is no evidence of coating damage or occurrence of corrosion on the copper substrate. The general trend regarding acid concentration is that the current density values are slightly higher at pH 1.4 than at pH 2.

### 3.3. Electrochemical Impedance Spectroscopy

**Figure 5:**
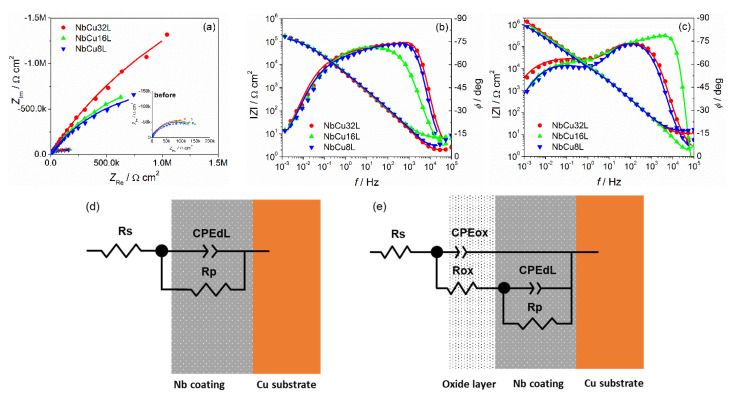
Electrochemical impedance spectra of Nb coatings on Cu in O_2_-saturated 0.05 M H_2_SO_4_ + 0.1 ppm F^−^ (pH = 1.4) solution at open circuit potential, at 90 °C: (**a**) Nyquist and (**b**) Bode plots before and (**c**) after polarization at constant potential *E* = 2 V for 6 h; (**d**) single time constant EEC and (**e**) two time constants EEC. Symbols are experimental data and continuous lines are simulated by fitting to the EEC.

Electrochemical impedance measurements were performed comparatively on metallic Nb and on the Nb coatings on Cu, at their OCP values, before and after passivation at a constant potential of 2 V for 6 h. Nyquist and Bode plots on metallic Nb (Figure S2 of the supporting information) exhibit a single capacitive loop and a single time constant, respectively, both before and after passivation. After passivation, the loop diameter increases from 30 to 90 kΩ cm^2^ due to the formation of a passive oxide layer on the surface. The complex plane plots of Nb coatings on Cu obtained at pH = 1.4 and given in Figure 5a, display a distorted capacitive loop before polarization, followed by an important increase in the impedance after polarization. Bode plots in Figure 5b,c show about a one order magnitude increase in the absolute impedance after polarization of Nb coatings. Phase angle plots are more sensitive in detecting time constants than complex plane plots. Consequently, Figure 5b,c point to the existence of two time constants, as indicated by the presence of two maxima both before and after polarization. Before polarization, a maximum at a frequency of 10^3^ Hz and a shoulder at 10^−1^ Hz are observed, which than develop into two well-defined time constants after polarization, with maxima at 10^2^ and 4 × 10^−2^ Hz. A different behavior was observed for Nb coatings at a higher pH. In this case, the presence of two time constants was evident only after polarization at 2 V for 6 h and only one time constant was present before polarization.

To model the behavior of metallic Nb and of Nb coatings on Cu, two equivalent electrical circuits were used, as depicted in Figure 5d,e. The first model is a single time constants EEC, which contains the solution resistance *R_S_* in series with a parallel connection of double-layer capacitance and the polarization resistance *R_P_*. The ideal capacitance was replaced by a constant phase element (CPE) to account for the non-ideality of the surface, with the impedance of CPE given by Equation (3):

(3) $$Z_{CPE}=\frac{1}{T{\left(j\omega \right)}^{n}}$$

where *T* is a parameter related to the double-layer capacitance according to Equation (4) and *n* is the parameter between 0 and 1 describing the constant phase angle of the CPE, which is n * 90°:

(4) $$T=C_{dl}^{n}{\left(R_{s}^{-1}+R_{p}^{-1}\right)}^{1-n}$$

The second model is two time constants EEC, to account for the presence of the passive oxide layer on the surface of Nb, including additionally the capacitance (CPE_ox_) and resistance (*R*_ox_) of the passive oxide layer. In this model, the high frequency time constant corresponds to the electrolyte/oxide layer interface and the low frequency time constant to the Nb-coating. The model in Figure 5d fitted very well the EIS results of metallic Nb and Nb coatings at pH = 2 before polarization and the model in Figure 5e was used to fit the impedance data of Nb coatings at pH = 2 after polarization and at pH = 1.4, both before and after polarization. The appearance of two time constants even before polarization at 2 V can be explained because the passive oxide layer on the surface of the Nb coatings is present to some extent even before polarization at 2 V, since it forms spontaneously in the air and its thickness increases during stabilization of OCP values in O_2_-saturated solutions at temperatures of 90 °C. The presence of only one time constant at pH = 2 before polarization is probably related to very low thickness of the passive oxide film.

The experimental EIS data of metallic Nb and Nb coatings were fitted to the corresponding EEC and the obtained values of the circuit elements are given in Table 4, together with their standard errors, the quality of fit expressed by the χ^2^ value and the calculated double layer capacitance values. It can be observed that both polarization resistance and resistance of the passive oxide layer increase after polarization at 2 V for 6 h.

Considering that the passive oxide layer acts as a dielectric between the electrolyte and the Nb coating, its capacitance can be expressed according to Equation (5):

(5) $$C_{ox}=\frac{\varepsilon \varepsilon_{0}A}{d_{ox}}$$

where *ε* is the relative dielectric constant, *ε*_o_ is the permittivity of vacuum (8.85 × 10^−14^ F cm^−1^), *A* is the effective surface area, and *d*_ox_ is the thickness of the oxide layer. The dielectric constant of amorphous anodic oxide films on niobium was reported to be 41 [47].

The effective surface area was taken as the surface fraction of Nb estimated by FE-SEM image processing. Consequently, the thicknesses of the passive films formed before/after polarization at 2 V for 6 h were estimated to be 1.0 nm/6.7 nm (metallic Nb), 2.5 nm/9.5 nm (NbCu8L), and 2.5 nm/10.9 nm (NbCu16L and 2.7/13.0 nm (NbCu32L)), respectively. These results are consistent with literature data [32], which indicate a thickness of about 1 nm for the air-formed natural oxide film and about 10 nm at 2 V for anodically grown oxide films on the Nb surface after fine mechanical polishing.

The values of polarization resistances determined by modeling the impedance date are of the same order of magnitude with values obtained by the Tafel extrapolation method (Table 5).

### 3.4. Post-Test Analytic

**Figure 6:**
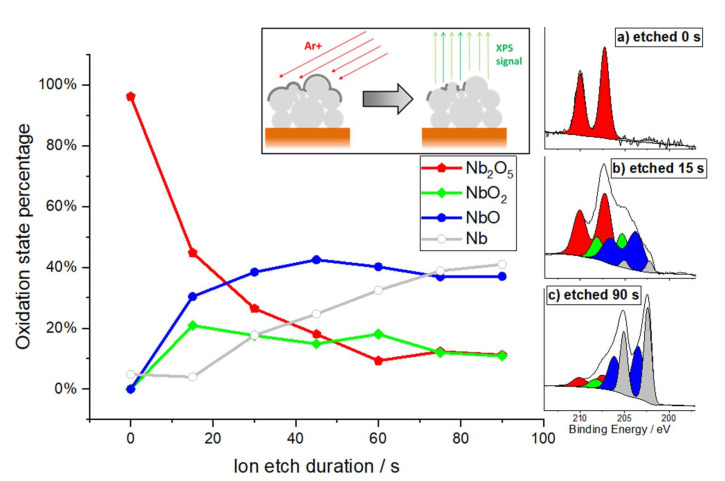
XPS depths profile of the oxide layer: The oxide layer of the Niobium coating is etched/reduced under Ar^+^ ion beam until steadiness is reached after ~90 s. Some areas of the grainy porous surface are hidden from the beam and lead to a remaining oxide signal. High resolution XPS spectrum after etching times of: (**a**) 0 s; (**b**) 15 s and (**c**) 90 s.

After the corrosion measurements, further analytics were performed on the NbCu8L sample. To investigate the composition of the formed oxide layers, XPS spectra were recorded. The chemical analysis of the uppermost surface of the Nb coating shows the clear predominance of niobium pentoxide, Nb_2_O_5_, the most stable oxide of this metal. The high-resolution photoemission spectrum of the Nb3d electron in Figure 6, insert (a) shows the dominant Nb^5+^ signal (red) and a marginal Nb^0^ signal (grey, compare Figure 6, insert (c) for clarity). Based on the spectral data, the average thickness of this oxide layer was calculated to 9–10 nm, using the database by Seah and Dench [48] for estimating the inelastic mean free path. Note, that these figures are slightly overestimated when samples are not flat on a nanometer scale.

The sample was carefully etched in steps of 15 s under an Argon ion beam (2 kV, 0.2 µA/mm²) to profile the surface layer. The etch rate was estimated to ~0.14 nm/s, based on sputter yield calculations by Matsunami et al. [49]. As the sputter yield of this method is typically higher for oxygen atoms than for niobium atoms, the oxide layer is partially reduced during the etching process, and the interface between oxide layer and intrinsically metallic Nb becomes blurred. Furthermore, there are inner surfaces of the porous coating, where additional native oxide becomes exposed, and the off-axis angle of the Argon beam leads to a “shadow” effect of the etching process.

As the equilibrium between oxide and metal is about to settle after 75–90 s, the average thickness derived from this method can be calculated as 10.5–12.6 nm. The entire set of Nb3d detail spectra for all etching steps can be found in the supporting information in Figure S3. No traces of defects of the niobium coating could be found, and no contaminations were carried into the layer. A depth profile of longer etching steps that shows the integrity of the coating is also presented in the supporting information in Figure S4.

**Figure 7:**
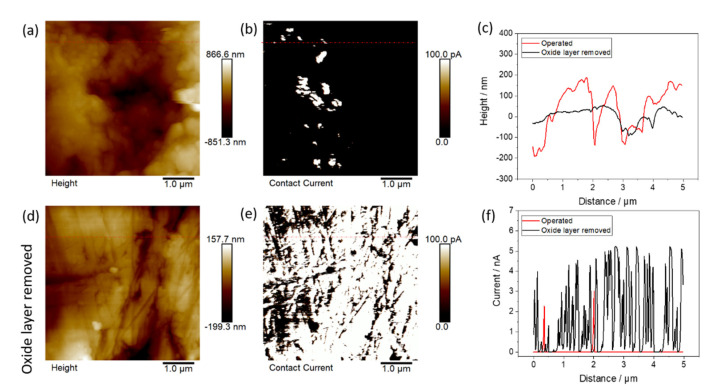
AFM measurements after corrosion tests of NbCu8L: (**a**) height of operated BPP, (**b**) electronic current of operated BPP, (**c**) height profiles of (**a**,**d**) as indicated by the red lines, (**d**) height of operated BPP (oxide layer removed), (**e**) electronic current of operated BPP (oxide layer removed) and (**f**) height profile of (**b**,**e**) as indicated by the red lines.

Additionally, conductive AFM measurements with 5 × 5 µm² on the surface of the Nb coating after the corrosion test revealed a low conductive area (3.4%) as seen in Figure 7b. The low conductive area concludes the growth of an oxide layer on the surface of the sample. The height image in Figure 7a shows the corresponding height measurement with an arithmetic average of the roughness profile (Ra) of 204 nm. In Figure 7c, the height profiles of one line each for the operated surface and the area with removed oxide layer are shown. To remove the oxide layer, the sample was grinded with sandpaper (4000). On the grinded part of the sample (Figure 7d), the conductive area increased to 84.6% (Figure 7e). This indicates that on the operated sample, an oxide layer with mostly nonconductive properties formed during the corrosion tests. The current profiles are shown in Figure 7f.

On the long-term operation of the BPP, the thickness and areal percentage of the oxide layer may increase and the conductivity on the nanoscale will decrease even further, resulting in a voltage increase over time for the whole cell. Nevertheless, the same is true for Ti-based BPP. To evaluate the increase in low conductive species over time, conductive AFM measurements could be performed at different time intervals of electrolyzer operation while collecting data cubes over a wide voltage range of large areas in future research.

### 3.5. PEMWE Test

**Figure 8:**
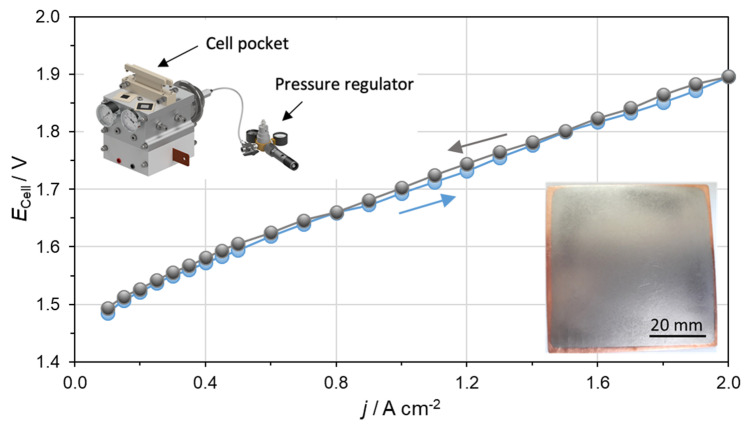
Polarization curve obtained using Nb-coated Cu pole plates, recorded up to 2 A cm^−2^ at 80 °C and 8 bar hydraulic pressure. The left inset shows the PEM electrolyzer cell produced by ProPuls used for the test. The right inset shows the Nb-coated Cu pole plate used in the cell.

Finally, an Nb-coated Cu pole plate was evaluated in a PEM electrolyzer with an active area of 25 cm^2^ as described in Section 2.5 to prove the ability of use in an operating system. Figure 8 presents the *E*_Cell_ *– j* characteristic curves up to a current density of 2 A cm^−2^ using a prototype testing cell from ProPuls, which is shown in the inset.

The reached performance of 1.90 V at 2 A cm^−2^ is within the range of published cell performances since 2010 [12,16] and comparable to performances of commercial electrolyzers on the market, such as Siemens [50]. The evaluation of the reported performance shows that the Nb-coated Cu pole plates can be operated on the anode side in a PEM electrolyzer and compared with state of the art technology. Polarization curves at current densities higher than 2 A cm^−2^ (here not shown) reveal a deviation between the ascending and descending curve. This behavior indicates mass transport limitations, which are related to inefficient transport of feed water from the porous transport layers towards the particular electrode surface as well as removal of product gases [26,51]. Further evaluation with electrochemical impedance spectroscopy would give detailed insight to the cell limitations. In conclusion, it can be said that the Nb-coated Cu pole plates are available for a long-term test in a PEM electrolyzer to confirm the long-term stability during operation. This can be seen as the final step towards commercialization.

## 4. Conclusions

We have reported the replacement of titanium bipolar plates by a low-cost material with superior electrical and thermal properties, such as copper coated with niobium. We have evaluated the performance of the anti-corrosive protection layers based on niobium applied by vacuum plasma spraying on copper pole plates. Coatings deposited by VPS are defect free and show very good adhesion to the copper substrate. Coatings with tailored thickness can be produced by varying the number of VPS layers, thus thicknesses of around 30, 70, and 130 µm were achieved. All coatings were tested according to a similar corrosion protocol, which included an accelerated stress test at a constant potential of 2 V applied for 6 h in a simulated PEM electrolysis environment at 90 °C and in O_2_-saturated solutions. Based on corrosion parameters determined by the Tafel extrapolation method, it was concluded that reduction of coating thickness from 130 µm to 30 µm preserves the anti-corrosion properties of the Nb layer, as proven by corrosion currents of about 0.1 µA cm^−2^. After polarization at 2 V, the presence of a passive oxide layer on the surface is evidenced by electrochemical impedance measurements, which show an increase in the polarization resistance. Both EIS and XPS analysis gave a similar estimate of the protective oxide layer thickness of about 10 nm, which is in the expected range for anodically oxidized Nb. The chemical composition determined by XPS shows the predominance of Nb_2_O_5_ in the outermost layer, but NbO_2_ and NbO were also detected. Finally, the Nb-coated copper pole plates were successfully operated in a PEM electrolyzer, which is a further step towards commercialization.

The results demonstrate the possibility to reduce production costs of titanium bipolar plates in PEM water electrolyzers by using alternative, low-cost materials, such as copper, and by substituting precious metal coatings with highly corrosion-resistant coatings based on niobium.

## Figures and Tables

**Table 1:** Chemical composition of resin and hardener used for sealing Nb coatings.

Chemical Composition	Weight
Resin	
Phenol, polymer with formaldehyde, glycidyl ether	60–90%
Alkyl (C12–14) glycidyl ether	5–15%
Oxirane, [((2-ethylhexyl)oxy)methyl]	0–10%
Bisphenol A–Epichlorohydrin polymer	0–5%
Hardener	
1,2-Cyclohexanediamine	40–70%
Poly[oxy(methyl-1,2-ethanediyl)], alpha-hydro-omega-(2-aminomethylethoxy)- ether with 2-ethyl-2-(hydroxymethyl)-1,3-propanediol (3:1)	20–50%
2,2′,2″-nitrilotriethanol	0–5%
Piperazine	0–2%

**Table 2:** Corrosion parameters of metallic Nb and Nb coatings on Cu in 0.005 M H_2_SO_4_ + 0.1 ppm F^−^ (pH = 2) before and after polarization at 2 V for 6 h.

Corrosion Parameters	Nb Metallic		NbCu8L		NbCu16L		NbCu32L	
	Before	After	Before	After	Before	After	Before	After
*E*_corr_ (V)	0.146	0.220	0.196	0.362	0.217	0.413	0.126	0.243
*j*_corr_ (µA cm^−2^)	1.20	0.15	0.064	0.018	0.078	0.023	0.021	0.015
*b*_a_ (mV decade^−1^)	194	362	151	227	162	189	164	199
*b*_c_ (mV decade^−1^)	−269	−121	−162	−112	−163	−126	−111	−115
*R*_P_ (kΩ cm^2^)	40.8	262.5	530.2	1809.2	452.3	1427.2	1473.3	1956.8

**Table 3:** Corrosion parameters of metallic Nb and Nb coatings on Cu in 0.05 M H_2_SO_4_ + 0.1 ppm F^−^ (pH = 1.4) before and after polarization at 2 V for 6 h.

Corrosion Parameters	Nb Metallic		NbCu8L		NbCu16L		NbCu32L	
	Before	After	Before	After	Before	After	Before	After
*E*_corr_ (V)	0.278	0.383	0.335	0.415	0.303	0.460	0.240	0.407
*j*_corr_ (µA cm^−2^)	1.23	0.45	0.133	0.054	0.092	0.029	0.070	0.024
*b*_a_ (mV decade^−1^)	176	326	184	342	140	296	163	259
*b*_c_ (mV decade^−1^)	−289	−270	−173	−121	−165	−99	−141	−98
*R*_P_ (kΩ cm^2^)	38.6	142.5	291.1	718.7	357.5	1110.8	469.0	1286.3

**Table 4:** EEC parameters obtained by fitting EIS data of metallic Nb and Nb-coatings in O_2_-saturated 0.05 M H_2_SO_4_ + 0.1 ppm F^−^ (pH = 1.4) solution at open circuit potential, at 90 °C.

Parameter	Nb Metallic		NbCu8L		NbCu16L		NbCu32L	
	Before	After	Before	After	Before	After	Before	After
R_S_ (Ω)	11.4 (0.4%)	10.5 (1.0%)	2.9 (2.2%)	14.0 (0.6%)	6.5 (1.5%)	10.1 (11.7%)	2.0 (1.9%)	10.4 (0.9%)
CPE-T_ox_ (F cm^−2^ s^n−1^)	1.08 × 10^−4^ (0.4%)	1.74 × 10^−5^ (0.8%)	8.70 × 10^−6^ (2.0%)	9.65 × 10^−6^ (1.5%)	4.12 × 10^−5^ (5.8%)	1.08 × 10^−5^ (1.5%)	9.73 × 10^−6^ (1.8%)	1.01 × 10^−5^ (1.7%)
*n*_ox_	0.86 (0.1%)	0.88 (0.2%)	1 (fixed)	0.86 (0.2%)	0.85 (0.8%)	0.85 (0.5%)	1 (fixed)	0.85 (0.3%)
*C*_ox_ (F cm^−2^)	3.63 × 10^−5^	5.37 × 10^−6^	8.70 × 10^−6^	2.26 × 10^−6^	9.44 × 10^−6^	2.18 × 10^−6^	9.73 × 10^−6^	2.01 × 10^−6^
*d*_ox_ (nm)	1.0	6.7	2.5	9.5	2.5	10.9	2.7	13.0
*R*_ox_ (kΩ cm^2^)	31.6 (0.6%)	92.8 (0.9%)	0.07 (19.1%)	9.6 (4.6%)	0.05 (fixed)	17.3 (21.0%)	0.06 (16.7%)	12.9 (7.5%)
CPE-T_dl_ (F cm^−2^ s^n−1^)	-	-	5.14 × 10^−5^ (1.2%)	1.81 × 10^−5^ (0.9%)	1.71 × 10^−5^ (13.7%)	1.21 × 10^−5^ (5.7%)	5.39 × 10^−5^ (1.2%)	1.13 × 10^−5^ (1.5%)
*n_dl_*	-	-	0.67 (0.6%)	0.63 (0.7%)	0.47 (8.5%)	0.63 (4.1%)	0.69 (0.6%)	0.65 (0.7%)
*R*_P_ (kΩ cm^2^)	-	-	175.8 (2.6%)	2110.0 (5.8%)	223.4 (7.7%)	2744.2 (25.2%)	195.4 (2.9%)	6948.4 (9.1%)
*χ*2	5.4 × 10^−4^	2.5 × 10^−3^	5.2 × 10^−3^	5.8 × 10^−4^	2.1 × 10^−3^	1.7 × 10^−2^	5.1 × 10^−3^	8.4 × 10^−4^

**Table 5:** Polarization resistance values determined by Tafel extrapolation and EIS fitting for metallic Nb and Nb coatings on copper in O_2_-saturated solutions at pH = 2 and pH = 1.4.

Sample	*R*_P-Tafel_ (kΩ cm^2^)				*R*_P-EIS_ (kΩ cm^2^)			
	Before pH = 2	After pH = 2	Before pH = 1.4	After pH = 1.4	Before pH = 2	After pH = 2	Before pH = 1.4	After pH = 1.4
Nb metallic	40.8	262.5	38.6	142.5	30.8	259.4	31.6	92.8
NbCu8L	530.2	1809.2	291.1	718.7	366.4	1913.6	175.8	2110.0
NbCu16L	452.3	1427.2	357.5	1110.8	262.5	4537.6	223.4	2744.2
NbCu32L	1473.3	1956.8	469.0	1286.3	1459.4	9008.4	195.4	6948.4

## Data Availability

Not applicable.

## Supplementary Materials

The following are available online at https://www.mdpi.com/article/10.3390/ma15051628/s1, Figure S1: Open circuit potential evolution in time for metallic Nb and Nb-coatings on copper in 0.005 M H_2_SO_4_ + 0.1 ppm F^−^ (pH = 2) and 0.05 M H_2_SO_4_ + 0.1 ppm F^−^ (pH = 1.4) measured in O_2_-saturated solution at 90 °C; Figure S2: Nyquist (a) and Bode plots (b) before and after polarization at constant potential *E* = 2 V for 6 h of metallic Nb in O_2_-saturated 0.05 M H_2_SO_4_ + 0.1 ppm F^−^ (pH = 1.4) solution at open circuit potential at 90 °C (symbols are experimental data and continuous lines are simulated by fitting to the EEC shown as insert); Figure S3: Ar^+^ etching steps of the Nb coating (15 s each) and the evolution of the occurring suboxide and metallic species, following with high-resolution XPS of the Nb3d region; Figure S4: Ar^+^ ion etching depth profile with long time scales.
