# Supplementary material for: Towards Replacing Titanium with Copper in the Bipolar Plates for Proton Exchange Membrane Water Electrolysis
Source: Materials (Basel). 2022 Feb 22;15(5):1628. doi: 10.3390/ma15051628 (PMC8911232; doi:10.3390/ma15051628)
Supplement: Supplementary file 1 [file materials-15-01628-s001.zip › materials-1584599-supplementary.pdf]

materials

MDPI

Supplementary

Towards Replacing Titanium with Copper in the Bipolar Plates for Proton Exchange Membrane Water Electrolysis

Andrea Kellenberger [1,*], Nicolae Vaszilcsin [1], Delia Duca [1], Mircea Laurentiu Dan [1], Narcis Duteanu [1], Svenja Stiber [2], Tobias Morawietz [2,3], Indro Biswas [2], Syed Asif Ansar [2], Pawel Gazdzicki [2], Florian J. Wirkert [4], Jeffrey Roth [4], Ulrich Rost [4,†], Michael Brodmann [4], Aldo Saul Gago [2,*] and K. Andreas Friedrich [2]

Electrochemical data

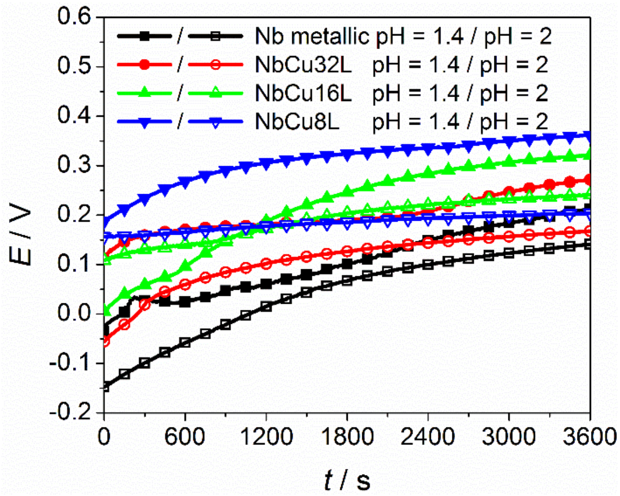

Figure S1. Open circuit potential evolution in time for metallic Nb and Nb-coatings on copper in 0.005 M H_2SO_4 + 0.1 ppm F^- (pH = 2) and 0.05 M H_2SO_4 + 0.1 ppm F^- (pH = 1.4) measured in O_2 saturated solution at 90°C.

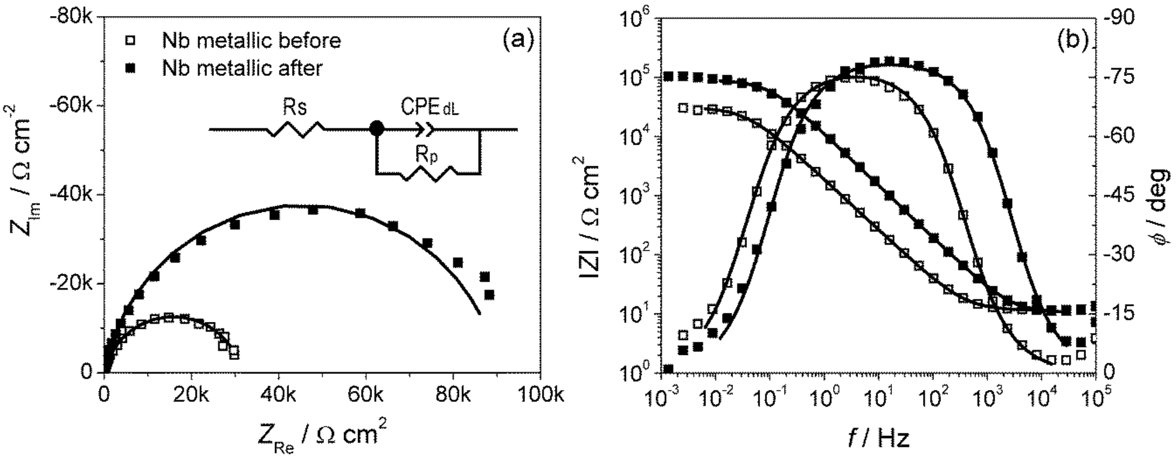

Figure S2. Nyquist (**a**) and Bode plots (**b**) before and after polarization at constant potential *E* = 2 V for 6h of metallic Nb in O_2 saturated 0.05 M H_2SO_4 + 0.1 ppm F^- (pH = 1.4) solution at open circuit potential, at 90°C. Symbols are experimental data and continuous lines are simulated by fitting to the EEC shown as insert.

Photoemission data

The sample NbCu8L was carefully etched in steps of 15 seconds under an Argon ion beam (2 kV, 0.2 µA/mm²) to profile the surface layer. The etch rate was estimated to ~0.14 nm/s. Each etching step was checked with a high resolution photoemission spectrum of the most prominent Nb 3d region.

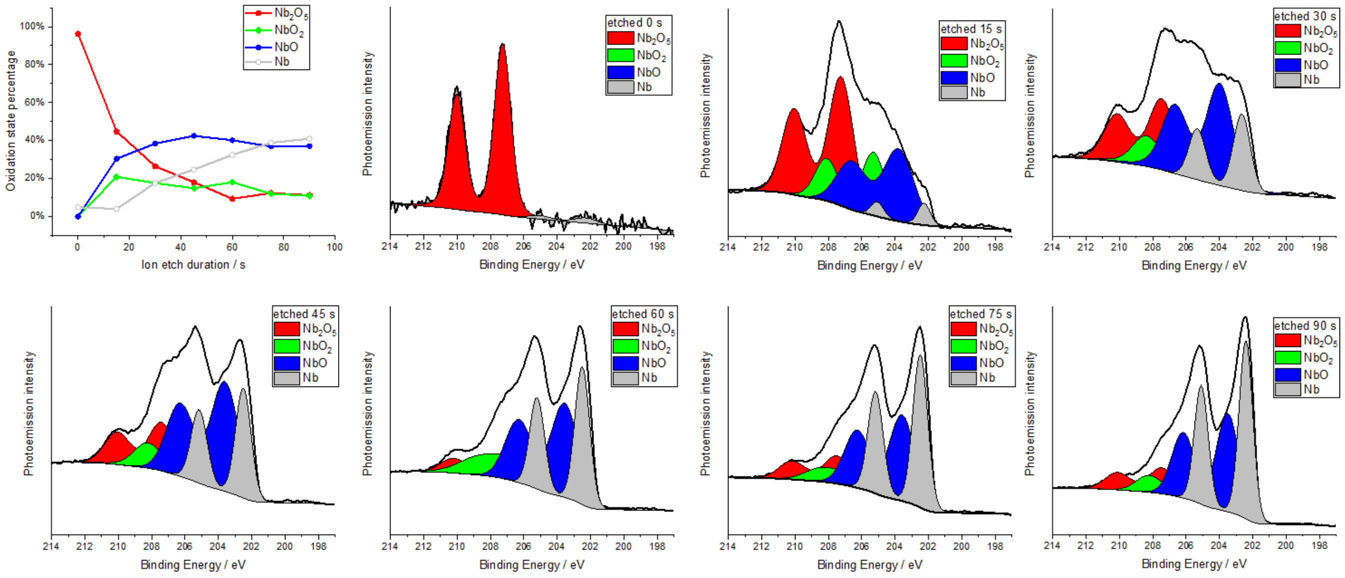

Figure S3. Ar^+ etching steps of the Nb coating (15 s each) and the evolution of the occurring suboxide and metallic species, following with high resolution XPS of the Nb3d region.

Figure S3 shows the individual steps. The colours indicate the four identified species with oxidations states of +V (red), +IV (green), +II (blue), and 0 (grey). The composition, which is summarized in the evolution graph in the first panel, reaches a steady state after about 75 seconds. Note, that the signal contains oxide species after sputtering, because of the shadowing effects of the used off-axis ion source, as the beam does not reach all areas of the grainy surface of this sintered material.

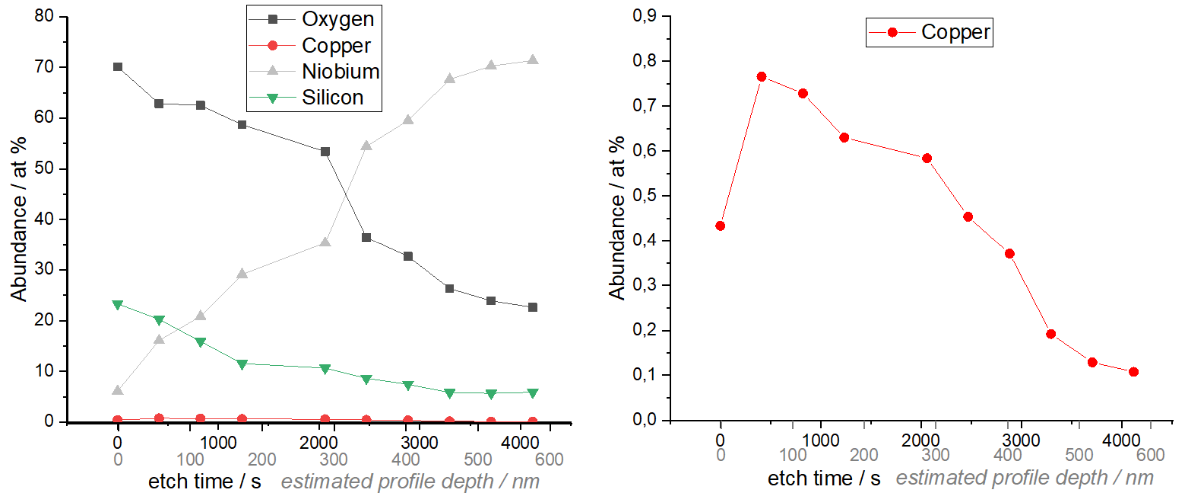

Figure S4. Ar^+ ion etching depth profile with long time scales.

A longer depth profile of the sample was recorded with identical etching conditions. The abundance of surface contaminations was traced with this method. Predominantly, a silicon signal and an oxygen signal are dominating the spectra during the first etching steps, due to the presence of silica from the mechanical surface treatment. The oxygen curve (black) is coarsely following the slope of the silicon curve, but is higher than the 2:1 ratio of these atoms in SiO_2, as it is expected from the sandpaper treatment before the corrosion tests, would suggest. As the native oxide layer of Nb_2O_5 of the Nb coating is also removed/reduced during the etching process, there is another contribution to this signal such as this oxygen from the epoxy-resin used to fill the pores. The niobium curve (grey) is steadily rising, as the metallic coating, which has been covered with a layer of oxide and contaminants in the nanometer scale before, is more and more exposed. A small amount of copper in the range of <1 at% was detected, which is steadily reducing with depth until the detection limit. The predominance of this element at the surface, within the first ~300 nm indicate, that this contamination does not have its source in the copper substrate – a diffusion through the coating would typically lead to an opposite slope, with the content steadily rising towards the substrate. Apparently, origin of the copper was on the surface of the niobium coating. It was reconstructed, that during the mechanical processing of the sample tiny grains of copper (of the substrate) have been distributed on top of the coating.
